# Donors With a Prior History of Cancer: Factors of Non-Utilization of Kidneys for Transplantation

**DOI:** 10.3389/ti.2023.11883

**Published:** 2023-10-31

**Authors:** Wai H. Lim, Eric Au, Armando Teixeira-Pinto, Esther Ooi, Helen Opdam, Jeremy Chapman, David W. Johnson, John Kanellis, Christopher E. Davies, Germaine Wong

**Affiliations:** ^1^ Medical School, University of Western Australia, Perth, WA, Australia; ^2^ Department of Renal Medicine, Sir Charles Gairdner Hospital, Perth, WA, Australia; ^3^ Department of Renal Medicine, Westmead Hospital, Sydney, NSW, Australia; ^4^ Sydney School of Public Health, University of Sydney, Sydney, NSW, Australia; ^5^ Centre for Kidney Research, Kids Research Institute, The Children’s Hospital at Westmead, Sydney, NSW, Australia; ^6^ School of Biomedical Sciences, University of Western Australia, Perth, WA, Australia; ^7^ DonateLife, Organ and Tissue Authority, Canberra, NSW, Australia; ^8^ Department of Intensive Care, Austin Health, Melbourne, VIC, Australia; ^9^ Centre for Transplant and Renal Research, Westmead Hospital, Sydney, NSW, Australia; ^10^ Princess Alexandra Hospital, Metro South Integrated Nephrology and Transplant Services, Brisbane, QLD, Australia; ^11^ Faculty of Medicine, University of Queensland, Brisbane, QLD, Australia; ^12^ Translational Research Institute, Brisbane, QLD, Australia; ^13^ Department of Nephrology, Monash Health, Melbourne, VIC, Australia; ^14^ Centre for Inflammatory Disease, Monash University, Melbourne, VIC, Australia; ^15^ Australia and New Zealand Dialysis and Transplant Registry, South Australian Health and Medical Research Institute, Adelaide, SA, Australia; ^16^ Adelaide Medical School, University of Adelaide, Adelaide, SA, Australia

**Keywords:** donor cancer, kidney donation, registry-based study, allograft failure, utilization

## Abstract

Cancer transmission from deceased donors is an exceedingly rare but potentially fatal complication in transplant recipients. We aimed to quantify the likelihood of non-utilization of kidneys for transplantation from donors with a prior cancer history. We included all intended and actual deceased donors in Australia and New Zealand between 1989 and 2017. Association between prior cancer history and non-utilization of donor kidneys was examined using adjusted logistic regression. Of 9,485 deceased donors, 345 (4%) had a prior cancer history. Of 345 donors with a prior cancer history, 197 (57%) were utilized for transplantation. Donor characteristics of age, sex and comorbidities were similar between utilized and non-utilized donors with prior cancer. The time from cancer to organ donation was similar between utilized and non-utilized donors, irrespective of cancer subtypes. Donors with a prior cancer history were less likely to be utilized [adjusted OR (95% CI) 2.29 (1.68–3.13)] than donors without prior cancer. Of all actual donors, the adjusted OR for non-utilization among those with prior cancer was 2.36 (1.58–3.53). Non-melanoma skin cancer was the most frequent prior cancer type for utilized and non-utilized potential donors. Donors with prior cancers were less likely to be utilized for transplantation, with no discernible differences in cancer characteristics between utilized and non-utilized donors.

## Graphical Abstract

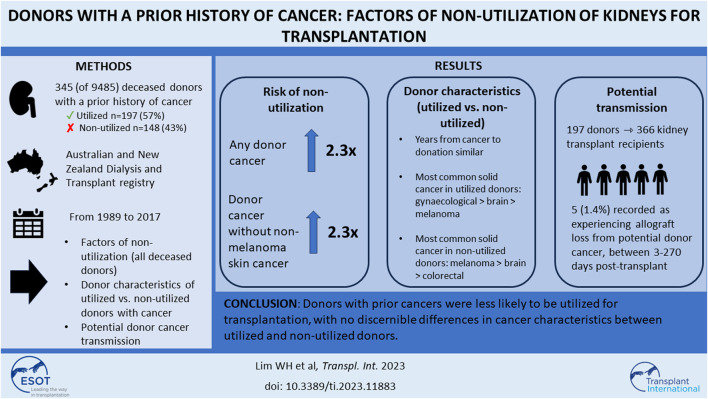

## Introduction

The ongoing shortage of donor organs to match the increasing demand has prompted organ donor programs to consider higher risk donors, such as those with a prior cancer history, for transplantation. Acceptance of organ donors with a cancer history for transplantation may vary according to recipient characteristics, the expected recipient survival after transplantation and types and stages of prior donor cancer(s).

Donor cancer transmission is an infrequent but potentially life-threatening complication in kidney transplantation. The risk of disease transmission is rare, but the frequency of donor-transmitted cancer is difficult to quantify because of potential reporting bias, and granular details of the donor history may be lacking at the time of procurement. Registry-based studies have reported the frequency of donor-transmitted cancers post-solid organ transplants was between 0 and 6 cases per 10,000 solid organ transplants, but varied according to the donor cancer type [1–8]. Prior research have found the most frequent donor-transmitted cancers were kidney cancers and melanomas. Once transmitted, the risk of death was highest among recipients of donor-transmitted lung cancer, melanoma and cancers of the central nervous system [6, 8–10]. However, these data are reliant on the published literature and registry analyses without knowledge of the specific details regarding the histological types, stages and treatment responses of these cancers. Knowledge of the reasons for non-utilization will provide guidance to assist clinicians and patients in future decision-making processes when considering transplantation of organs from these donors.

Given the ongoing uncertainty concerning the likelihood of cancer transmission from donors with prior cancers coupled with the significant patient morbidity and mortality associated with donor-transmitted cancers [9], there are considerable centre and country variations in clinical decision making regarding donor utilization for transplantation. In addition, the actual non-utilization rate of organ donors with cancer remains unclear. In this study, we aimed to determine the likelihood and factors of donor non-utilization. We also defined the risks and outcomes of transplant recipients who received kidneys from donors with a prior cancer history.

## Materials and Methods

### Study Population

All intended and actual deceased donors (i.e., consented for donation) in Australia and New Zealand between 1989 and 2017 from the Australia and New Zealand Organ Donation (ANZOD) and Australia and New Zealand Dialysis and Transplant (ANZDATA) registries were included in this study. For deceased donors with a past history of any cancers (including solid cancers, haematological cancers and non-melanoma skin cancers [NMSC]), data for these prior cancers were extracted from the registries. In addition, kidney transplant recipients (and their matched donors with prior cancer history) who had lost their kidney allografts from donor cancer were also identified from the registries. Data of allograft loss in kidney transplant recipients who have received deceased donor kidneys without a prior cancer history in the same time period were also extracted. An intended organ donor was a person for whom the donation work was initiated and a formal written consent for organ donation was undertaken, but did not become an actual donor (i.e., kidneys were not retrieved); whereas an actual donor was a person for whom the organ retrieval procedure had commenced for the purpose of transplantation, even if organs were not utilized for transplantation.

The clinical and research activities being reported are consistent with the Principles of the Declaration of Istanbul as outlined in the “Declaration of Istanbul on Organ Trafficking and Transplant Tourism.” This study was approved by the Human Research Ethics Committee of the University of Western Australia.

### Data Collection

Deceased donor characteristics included age, sex, ethnicity, primary cause of death, donation pathway [donation after neurological determination of death (DNDD) and donation after circulatory determination of death (DCDD)], comorbid conditions (diabetes, hypertension, smoking history and prior hepatitis C exposure), terminal estimated glomerular filtration rate (eGFR, in mL/min/1.73 m [2]) and donation era. Donor cancer details extracted from the registries included date of cancers, site(s), histology and treatment(s) (if data were available).

### Exposure

The study groups were categorized into non-utilized (either intended donors or actual donors whose kidneys were retrieved but were not utilized for transplantation) and utilized (actual donors whose kidneys were retrieved and were utilized for transplantation) donors.

### Study Outcomes

The primary outcome was the risk of non-utilization of the donor kidneys. If only one kidney was utilized (and the other kidney was not utilized), the donor was considered as being utilized. The characteristics of donors with prior cancer history were described, stratified by utilized and non-utilized donors. We also determined the risk and outcomes of cancer transmission among recipients who received kidneys from donors with known prior cancers. In this study, donor cancer transmission was defined as allograft loss reported to ANZDATA registry as being attributed to donor cancer. Consequently, allograft loss from donor cancer may include donor cancers that were transmitted at time of organ donation, cancers that were derived from donor cells and circumstances where a decision was made (by clinicians and recipients) to “terminate” allograft function following detection of donor cancer. The registry does not verify the accuracy of the reporting or require evidence that cancer cells were of donor origin, and it does not collect the exact reason for the reported allograft loss from donor cancer by the transplanting centres.

### Statistical Analyses

Baseline characteristics were expressed as number (proportion), median [interquartile range (IQR)] and mean (standard deviation, SD) where appropriate; with comparisons between utilized and non-utilized donors examined using chi-square test, Kruskal-Wallis test and t-test, respectively. The association between donor cancer history (with and without inclusion of donors with prior NMSC) and non-utilization was determined using logistic regression models, with the estimates expressed as unadjusted and adjusted odds ratio (OR) and 95% confidence intervals (95% CI). Covariates included in the multivariable models were selected according to known biological relationships with outcomes and included donor age, donor hypertension, donor diabetes status, donor smoking history, donor hepatitis C viral status [nucleic acid test (NAT)], donor ethnicity, donor terminal eGFR, donor death pathway (DNBD or DCDD) and era. Two sensitivity analyses were undertaken. First, characteristics of specific cancer subtypes were described and compared between 3 groups of intended donors, actual donors with and without kidneys utilized for transplantation. Second, an analysis restricting to actual donors (i.e., intended donors were excluded) was undertaken to examine the association between donor cancer history and non-utilization, with adjustment of donor covariates as the main model.

To define the risks and outcomes of donor cancer transmission, we compared the characteristics between donors that were utilized and those that were not utilized for transplantation. We focussed on pre-specified donor cancer types including melanoma, brain, breast, kidney and bladder, gynaecological, prostate and haematological cancers using descriptive analysis. We determined the risk of cancer transmission and subsequent allograft loss in recipients who received a donor with prior cancer history. We also described the donor and recipient characteristics of allograft loss from potential donor cancer in kidney transplant recipients who have received deceased donor kidneys without a prior cancer history. All analyses were undertaken using Stata (version 15.1 StataCorp LP, College Station, TX).

## Results

**FIGURE 1:**
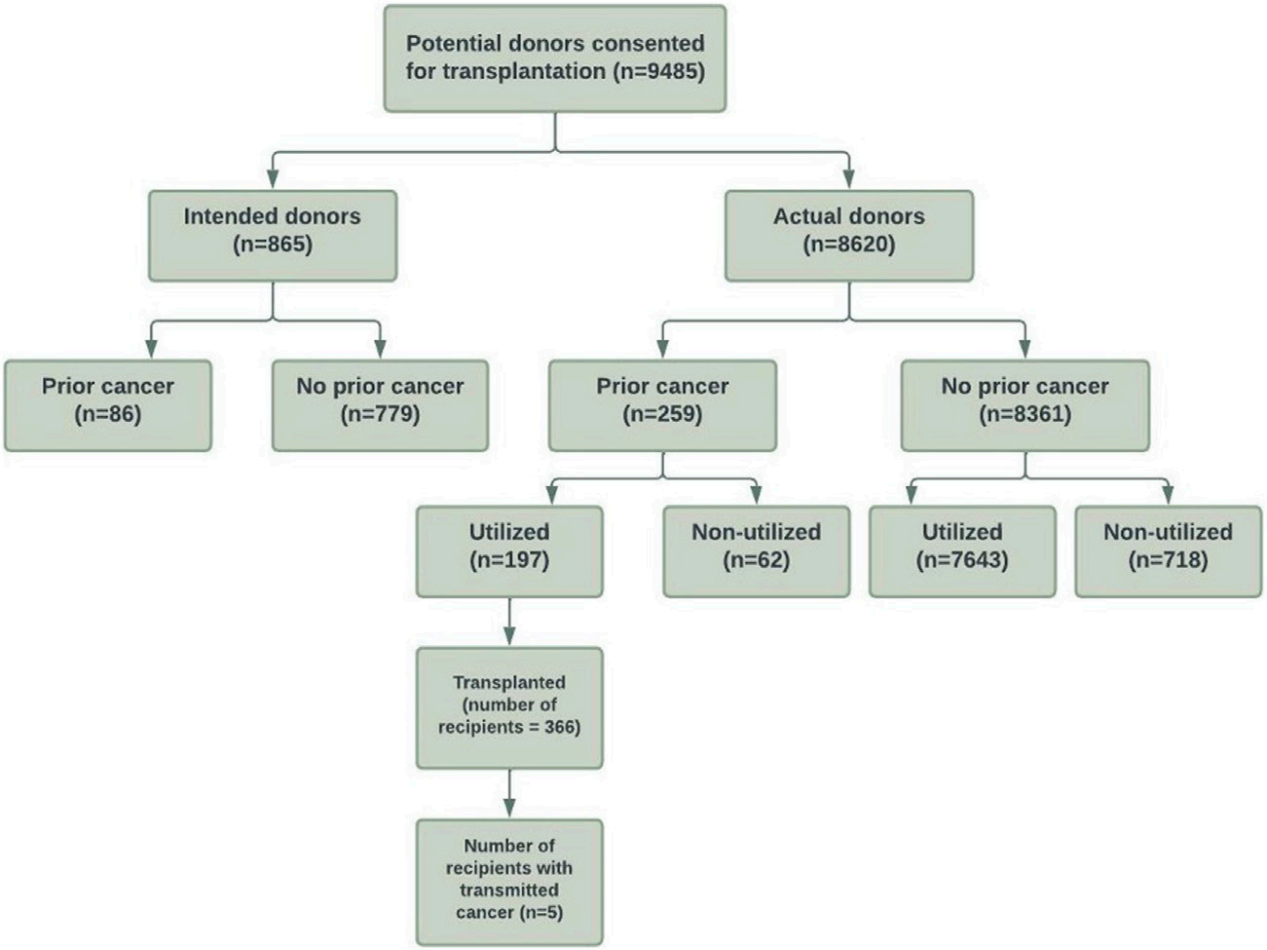
Flow diagram of the study cohort of consented intended and actual deceased donors with and without prior cancer history in Australia and New Zealand between 1989 and 2017.

The study cohort is shown in Figure 1. There were 9,485 intended and actual deceased donors, of these, 1,645 (17%) were not utilized for kidney transplantation. Table 1 shows the donor characteristics of kidneys from utilized and non-utilized donors. The mean (SD) age was higher (49 [18] vs. 41 [18]) and a greater proportion had diabetes (12% vs. 3%), were current smokers (42% vs. 38%), had a history of hypertension (38% vs. 20%) and positive hepatitis C virus NAT (5% vs. <0.1%) compared with utilized donors. The primary causes of donor death attributed to cerebral hypoxia/ischaemia or infarct was 37% in non-utilized donors compared to 19% in utilized donors.

**TABLE 1:** Baseline characteristics of the study cohort.

	Deceased donor utilization status		
	Non-utilized donors (*n* = 1,645)	Utilized donors (*n* = 7,840)	*p*-value
Donor characteristics			
Female (n, %)	647 (39.3)	3,351 (42.7)	0.01
Age (years, mean [SD])	49.5 (18.0)	41.5 (17.9)	<0.01
Ethnicity (n, %)			<0.01
White	1,460 (88.7)	7,283 (92.9)	
Australian Aboriginals/TSI	36 (2.2)	79 (1.0)	
Asians	100 (6.1)	297 (3.8)	
New Zealand Māoris	19 (1.2)	91 (1.2)	
Others	30 (1.8)	90 (1.1)	
Donor cancer history			
Any cancer (including NMSC)	148 (9.0)	197 (2.5)	<0.01
Any non-NMSC cancer	113 (6.9)	141 (1.8)	<0.01
Donor comorbid conditions			
BMI (kg/m^2^, mean [SD])	27.2 (6.1)	25.8 (5.7)	<0.01
Missing data (n, %)	64 (3.9)	375 (4.8)	
Diabetes (n, %)			<0.01
None	1,345 (81.8)	6,571 (83.8)	
Yes	198 (12.0)	264 (3.4)	
Missing data	102 (6.2)	1,005 (12.8)	
Hypertension (n, %)			<0.01
None	966 (58.7)	5,398 (68.9)	
Yes	615 (27.4)	1,376 (17.5)	
Unknown/missing data	64 (3.9)	1,066 (13.6)	
Smoking history (n, %)			<0.01
None	542 (32.9)	2,960 (37.8)	
Former	362 (22.0)	1,201 (15.2)	
Current	684 (41.6)	2,592 (33.1)	
Unknown/missing data	57 (3.5)	1,087 (13.9)	
Hepatitis C virus NAT (n, %)			<0.01
Negative	1,566 (95.2)	7,834 (99.9)	
Positive	79 (4.8)	6 (0.1)	
Donation pathway characteristics			
DCDD (n, %)	691 (42.0)	861 (11.0)	<0.01
Cause of death (n, %)			<0.01
Cerebral infarct/hypoxia	601 (36.5)	1,518 (19.4)	
Intracranial haemorrhage	621 (37.8)	3,401 (43.4)	
Traumatic brain injury	244 (14.8)	2,069 (26.4)	
Others	179 (10.9)	852 (10.8)	
Donor terminal eGFR [mL/min/1.73 m [2], mean (SD)]	78.8 (42.1)	93.6 (34.4)	0.78
Missing data (n, %)	780 (47.4)	132 (1.7)	
Era of donation (n, %)			<0.01
1989–1998	97 (5.9)	2,268 (28.9)	
1999–2007	228 (13.9)	1,932 (24.6)	
2008–2017	1,320 (80.2)	3,640 (46.5)	

Data expressed as number (%) or as mean [standard deviation (SD)]. TSI, Torres Strait Islander; eGFR, estimated glomerular filtration rate; NAT–nucleic acid test; DCDD, donation after circulatory determination of death; NMSC, non-melanoma skin cancer.

Of 8,620 actual donors, 780 (9%) were not utilized for transplantation. Non-utilized actual donors were more likely to have diabetes (12% vs. 3%), have a positive hepatitis C virus NAT (4 vs. <0.1%), and were more likely to be DCDD donors (17% vs. 11%) compared to utilized actual donors. A greater proportion of actual donors were not utilized for transplantation in the more recent era compared to the earlier eras (1989–1998: 4%, 1999–2007: 7%, 2008–2017: 13%).

### Donors With Prior Cancer History

Of the intended and actual donors, 345 (4%) donors had a prior history of cancers (254 [3%] donors with solid/haematological cancers and 91 [1%] with NMSC). Of these donors with prior cancer history, 197 (57%) donors were utilized for transplantation.

Of the actual donors, 259 (3%) donors had a prior history of cancers (190 [2%] donors with solid/haematological cancers and 69 [1%] with NMSC). Of these donors, 62 (24%) donors were not utilized. If restricted to donors with solid/haematological cancers, 49 of 190 (26%) donors were not utilized for transplantation. Supplementary Table S1 shows the cancer types of “Other” cancers.

### Association Between Donor Prior Cancer History and Non-utilization of Donor Kidneys

**FIGURE 2:**
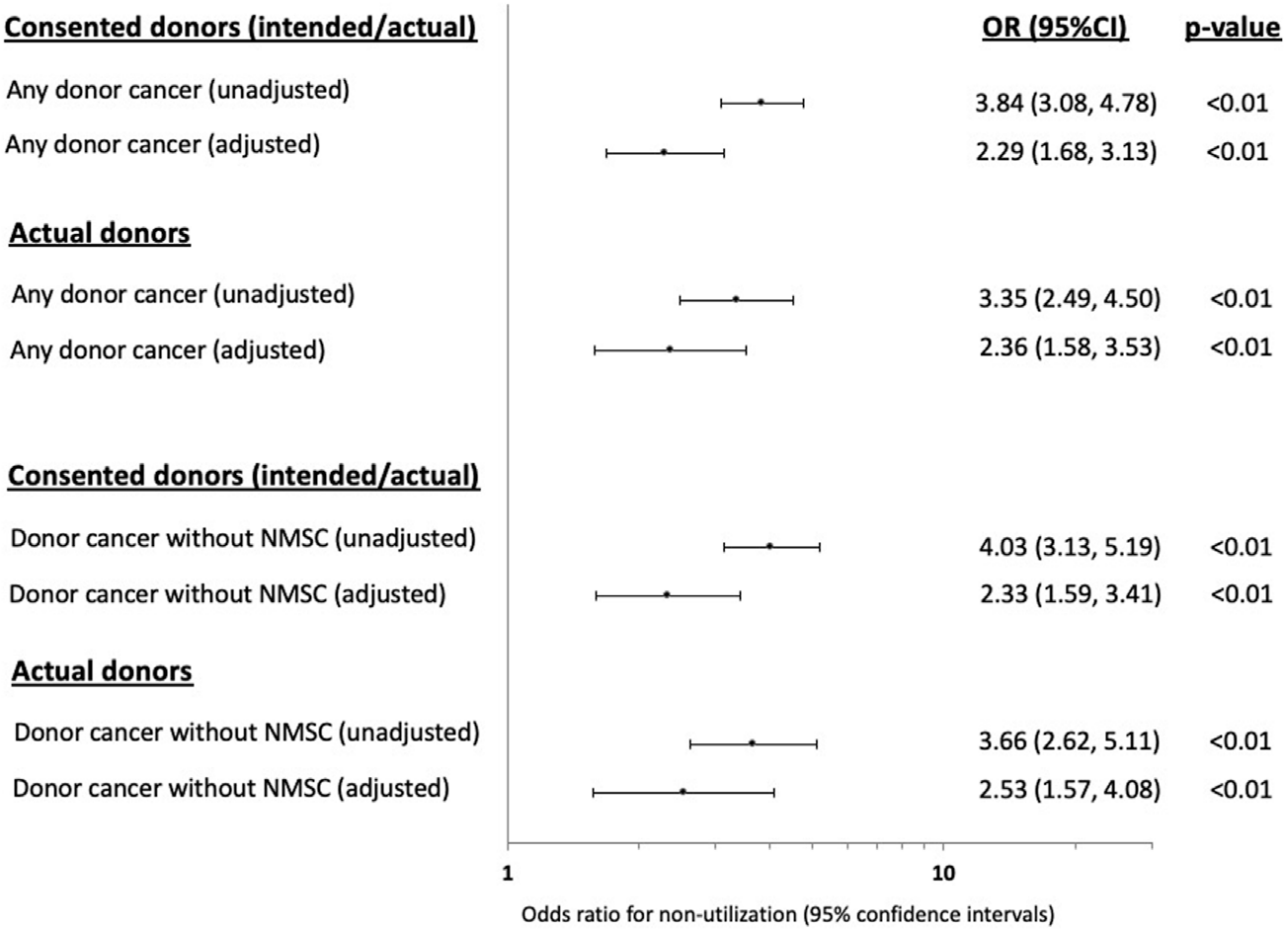
Forest plots showing the unadjusted and adjusted odds ratio (OR) with 95% confidence intervals (95% CI) of the associations between prior donor cancer history [with and without a prior history of non-melanoma skin cancer (NMSC)] and non-utilization of kidneys from intended and actual donors.

Compared to donors without a cancer history, the adjusted OR (95% CI) for non-utilization among donors with any prior cancer was 2.29 (1.68, 3.13). Other donor factors associated with an increased risk of non-utilization included older donor age, current smokers, DCDD donor status, prior history of donor hypertension or diabetes, lower terminal donor eGFR and positive donor hepatitis C NAT (Figure 2; Supplementary Table S2). The adjusted OR for non-utilization among donors with only a prior history of solid/haematologial cancers was 2.33 (1.59, 3.41).

In the sensitivity analysis restricting to actual donors (intended donors excluded, *n* = 865), the adjusted OR (95% CI) for non-utilization of donors with any prior cancer history was 2.36 (1.88, 3.53) and was 2.53 (1.57, 4.08) for donors with only a prior history of solid/haematologial cancers (Figure 2; Supplementary Table S2).

### Characteristics of Donors With Prior Cancer History

There were 345 (4% of study cohort) donors with a prior history of cancer, of these, 91 (26%) had a prior history of NMSC and 254 (74%) donors a prior history of solid organ or haematological malignancy (including malignant melanoma). The median (IQR) time from donor cancer diagnosis to consent for donation was 5.8 (0.7, 11.9) years for non-utilized intended/actual donors, and 7.5 (1.4, 16.2) years for utilized actual donors. Of the 91 donors with prior NMSC, kidneys from 56 (62%) donors were utilized for transplantation, respectively. Of the 254 donors with prior cancers other than NMSC, 113 (44%) donors were not utilized for transplantation. Table 2 shows the characteristics of the utilized and non-utilized donors for transplantation. The majority of the donors were of White backgrounds, and the proportion of donors with comorbidities was similar in both utilized and non-utilized groups. NMSC was the most frequent prior cancer type for both utilized and non-utilized potential donors. Among non-utilized potential donors, the most frequent prior cancer types were melanoma, colorectal, brain and prostate cancers.

**TABLE 2:** Characteristics of utilized and non-utilized deceased donors with prior cancer history.

		Non-utilized donors (*n* = 148)	Utilized donors (*n* = 197)	*p*-values
Donor demographics	Age (mean, SD)	58.9 (15.3)	56.9 (12.1)	0.171
	Age (median, IQR)	61.0 (52.0, 69.0)	60.0 (48.0, 67.0)	0.062
	Female (n, %)	66 (44.6)	107 (54.3)	0.074
	Race (n, %)			0.515
	White	141 (95.2)	193 (98.0)	
	Asian	4 (2.8)	2 (1.0)	
	Aboriginal	3 (2.0)	2 (1.0)	
	Comorbid condition (n, %)			
	Diabetes	19 (12.9)	13 (6.6)	0.129
	Hypertension	68 (45.9)	69 (35.0)	0.071
	Smoking (former/current)	80 (54.1)	122 (61.9)	0.400
	Hepatitis C virus NAT positive	2 (1.4)	0 (0.0)	0.384
Prior donor cancer history	Cancer types (n, %)			
	Melanoma	22 (14.9)	18 (9.1)	
	Brain	12 (8.1)	19 (9.6)	
	Colorectal	11 (7.4)	3 (1.5)	
	Haematological cancers	6 (4.1)	6 (3.0)	
	Breast	7 (4.7)	14 (7.1)	
	Thyroid gland	3 (2.0)	8 (4.1)	
	Female gynaecological	8 (5.4)	30 (15.2)	
	Prostate	11 (7.4)	14 (7.2)	
	Kidney/Bladder	10 (6.8)	11 (5.6)	
	Respiratory	2 (1.4)	1 (0.5)	
	NMSC	35 (23.6)	56 (28.4)	
	Others/unknown	21 (14.2)	17 (8.6)	
	Years from cancer to donation			
	Mean (SD)	8.7 (12.0)	11.0 (14.4)	0.113
	Median (IQR)	5.8 (0.7, 11.9)	7.5 (1.4, 16.2)	0.287
Donation history	Donor status (n, %)			<0.001
	Actual donor	62 (41.9)	197 (100.0)	
	Intended donor	86 (58.1)	0 (0.0)	
	“Intended” DNDD	88 (59.5)	155 (78.7)	<0.001
	Causes of death (n, %)			
	Intracranial haemorrhage	75 (50.7)	112 (56.9)	
	Cerebral infarct	12 (8.1)	20 (10.2)	
	Cerebral hypoxia/ischaemia	29 (19.6)	25 (12.7)	
	Traumatic brain injury	18 (12.2)	20 (10.2)	
	Others	9 (6.0)	19 (9.7)	
	Missing data	5 (3.4)	1 (0.3)	
	Year of donation (n, %)			0.437
	1989–1999	0 (0.0)	3 (1.5)	
	2000–2005	18 (12.2)	19 (9.6)	
	2006–2010	20 (13.5)	32 (16.3)	
	2011–2015	60 (40.5)	83 (42.1)	
	2016–2017	50 (33.8)	60 (30.5)	

Data expressed as number (%), mean [standard deviation (SD)] or as median [interquartile range (IQR)]. NAT, nucleic acid test; DNDD, donation after neurological determination of death; NMSC, non-melanoma skin cancer.

### Donor Cancer Characteristics in Selected Prior Cancer Types

Table 3 shows the cancer types and treatment strategies among potential donors with prior cancers, stratified by donor utilization status. The time from cancer occurrence to donor consent/organ donation varied by prior cancer types, but these time periods were similar for both utilized and non-utilized donors. The median (IQR) duration between time of cancer diagnosis to the time of consent for organ donation were 9.0 (4.9, 14.3) years for donors with prior colorectal cancer, 20.0 (10.3, 24.9) years for donors with prior breast cancer, 10.1 (5.4, 17.1) years for donors with prior melanoma, 4.1 (2.8, 8.0) years for donors with prior prostate cancer, 1.0 (<0.1, 9.6) years for donors with prior brain cancer and 13.0 (6.4, 20.7) years for donors with prior gynaecological cancers.

**TABLE 3:** Specific cancer subtypes of utilized and non-utilized donors with prior cancer history.

	Non-utilized donors (*n* = 148)	Utilized donors (*n* = 197)	*p*-values
Donor cancer types			
Melanoma (n)	22	18	0.79
Sites (n)			
Skin	20	16	
Non-skin	0	1	
Unknown	2	1	
Treatment			
Surgery	18	14	
None/Others	1	2	
Unknown	3	2	
Years to donation^a^	10.2 (5.4, 15.9)	10.1 (5.4, 28.5)	
Brain (n)	12	19	0.27
Types			
Astrocytoma	4	8	
Low-grade glioma	1	2	
High grade glioma/GBM	3	5	
Medulloblastoma	3	0	
Meningioma	1	0	
Others	0	4	
Treatment			
Surgery	9	5	
Radiotherapy	1	1	
Chemotherapy	0	1	
None	2	9	
Unknown/others	0	3	
Years to donation^a^	2.1 (0.1, 20.9)	0.7 (0.1, 8.8)	
Colorectal (n)	11	3	0.14
Types			
Adenocarcinoma	5	2	
Unknown	2	0	
Carcinoid	1	0	
Others	3	1 (carcinoma-in-situ)	
Treatment			
None	2	0	
Surgery	6	3	
Chemotherapy/radiotherapy	0	0	
Unknown/others	3	0	
Years to donation^a^	7.5 (3.7, 14.3)	14.1 (10.1, 26.7)	
Breast (n)	7	14	0.16
Types			
Adenocarcinoma	4	2	
Invasive ductal carcinoma	2	2	
Ductal carcinoma *in situ*	1	1	
Unknown	0	9	
Treatment			
None	1	0	
Surgery	4	11	
Chemotherapy/radiotherapy	1	1	
Unknown/others	1	2	
Years to donation^a^	10.3 (4.6, 21.7)	20.3 (17.8, 25.3)	
Prostate (n)	11	14	0.16
Types			
Adenocarcinoma	6	12	
Others	3	0	
Unknown	2	2	
Treatment			
None	2	1	
Surgery	6	10	
Chemotherapy/radiotherapy	1	2	
Unknown/others	2	1	
Years to donation^a^	3.1 (0.2, 7.9)	5.4 (3.3, 11.0)	
Kidney/bladder (n)	10	11	-
Types			
RCC	7	8	
Papillary cancer (kidney)	1	0	
Kidney oncocytoma	1	1	
Bladder (urothelial/TCC)	1	2	
Treatment			
None	9	8	
Surgery	1	2	
Unknown/others	0	1	
Years to donation^a^	-	0.8 (0.3, 1.1)	
Gynaecological (n)	8	30	0.22
Types			
Cervical cancer (SCC/adenocarcinoma)	5	13	
Cervical cancer *in situ*	0	0	
Uterine	0	2	
Others/Unknown	3	15	
Treatment			
None	0	1	
Surgery	4	22	
Chemotherapy/radiotherapy	1	0	
Unknown/others	3	7	
Years to donation^a^	21.4 (9.5, 24.8)	11.0 (6.4, 18.1)	
Haematological (n)	6	6	0.87
Types			
Leukaemia	1	2	
Lymphoma	5	4	
Treatment			
None	3	3	
Surgery	0	0	
Chemotherapy/radiotherapy	1	2	
Unknown/others	1	1	
Years to donation^a^	0.9 (-, 6.6)	3.6 (0.8, 16.5)	

^a^ Represents median [interquartle range (IQR)] years to donation using available recorded data. GBM, glioblastoma multiforme; RCC, renal cell cancer; DCIS, ductal carcinoma-in-situ, TCC, transitional cell cancer.

The treatment strategies for the prior cancers were similar between utilized and non-utilized actual donors; with majority of donors with prior melanoma, colorectal, breast, prostate and gynaecological cancers had surgery or chemo-radiotherapy. Among the 31 intended donors with prior brain cancers, 11 (35%) had high grade gliomas/glioblastoma multiforme or medulloblastoma. Of these 11 donors, 5 (45%) donors were utilized for transplantation. Of the 14 donors with colorectal cancer, 7 (50%) cancers were adenocarcinoma, of which 2 (29%) were utilized for transplantation. Of the 21 donors with breast cancer, 14 (67%) donors were utilized for transplantation. Of the 38 donors with gynaecological cancers, 30 (79%) donors were utilized for transplantation (of which 13 cancers were cervical cancers and 15 cancers were unknown/not reported).

In a sensitivity analysis examining the cancer characteristics of intended and utilized and non-utilized actual donors, times from donor cancer to organ donation were lower in intended donors compared to actual donors for melanoma, colorectal and breast cancer, but these were not statistically significant (Supplementary Table S3).

### Risk of Cancer Transmission in Donors With Prior Cancers

Three-hundred and sixty-six recipients received kidneys from 197 donors with a prior cancer history, with a median allograft follow-up period of 4 years. Of these recipients, 5 (1.4%) were recorded as experiencing allograft loss from four donors with prior cancer. All 4 donors died from intracranial haemorrhage. There were 2 donors with a prior history of non-Hodgkin lymphoma, 1 donor with prior malignant melanoma and 1 donor with prior kidney cancer. All 5 recipients were reported to be alive at the end of follow-up (31st December 2017), with allograft loss reported to occur between 3–270 days post-transplant. Donor kidney cancer transmission occurred in one recipient who received a kidney from a donor with a prior kidney cancer at 8 months post-transplant. Two recipients were reported to have lost their allografts from donor cancer but the type of cancer (in the allograft) was not recorded in the registry (Table 4).

**TABLE 4:** Characteristics of kidney transplant recipients with allograft loss attributed to donor cancer.

Donor cancer transmission (year)	Prior donor cancer (type)^a^	Donor age	Cause of donor death	Recipient cancer type/site (days from transplant if known)*	Time to allograft loss in days
1 (1998)^a^	Yes (NHL)	72	Intracranial haemorrhage	Lymphoma in allograft	11
2 (1998)^a^	Yes (NHL)	72	Intracranial haemorrhage	Lymphoma in allograft	10
3 (2001)	Yes (melanoma)	47	Intracranial haemorrhage	None	155
4 (2003)	Yes (renal cell cancer)	56	Intracranial haemorrhage	Adenocarcinoma in allograft (255)	270
5 (2008)	Yes (NHL)	68	Intracranial haemorrhage	None	3

^a^ Same donor (for cases 1&2). NHL, non-Hodgkin lymphoma. *Note all recipients remain alive at the end of survey period.

Over the study period, of the 14,671 kidney transplant recipients who have received kidneys from donors without a known prior cancer history, there were 12 recipients who were reported to experience potential donor cancer-related allograft loss from 10 deceased donors (i.e. 10/7,643 donors without a prior history of cancer at the time of organ donation or 0.1%). Intracranial haemorrhage was the cause of death in 5 donors. The exact cause of allograft loss (or whether a cancer was detected in the allograft) was not reported to the registry (Supplementary Table S4).

## Discussion

In this large registry study spanning almost three decades, we have shown that donors with a prior cancer history were less likely (by approximately 2-fold) to be utilized for kidney transplantation compared to donors who did not have prior cancer. While cancer history influenced the likelihood of utilization of consented donor kidneys, there were other important donor factors such as terminal kidney function and donor comorbidities that clinicians would consider for non-utilization. Although donors with a prior cancer history comprised of only 4% of all utilized donors, over 50% of these donors (with prior cancer history) were utilized for transplantation. The time from diagnosis to organ donation varied by cancer types, with average duration of between 4 (or less) years for brain, prostate and kidney cancers; and between 10 and 20 years for melanoma, colorectal and breast cancers. There were a total of 5 (1%) reported cases of donor cancer transmission over a median follow-up time of 4 years from 366 recipients who have received kidneys from 197 donors with a prior history of cancer, suggesting transplant clinicians follow a relatively conservative approach in accepting higher risk donors. Of those with transmitted disease, all recipients experienced allograft loss, with 3 of 5 cases reported to have cancer detected in the allograft. None of the recipients died as a result of cancer transmission. Based on the current dataset, we could only speculate donor origin cancers were either transmitted with the allograft or were derived from the allograft. However, given that allograft loss (attributed to donor cancer) in these 5 cases occurred early post-transplant (range 3–270 days), it is therefore less likely that these donor cancers (presumed to cause allograft loss) represent *de novo* (new) recipient-derived cancer.

The rate of non-utilization of donor organs for kidney transplantation varies between countries and donor quality, with rates of donor discards reported up to 20% [11, 12]. Several cohort studies have examined predictive factors for non-utilization and these included older donor age, female donors, hepatitis B and C seropositive status, higher terminal serum creatinine concentration and donor comorbidities, such as hypertension, diabetes and smoking history [13–15]. It is important to emphasize that the metrics and terminologies of utilization and non-utilization of donor kidneys are inconsistent across studies, and therefore reliable comparisons between regions and countries could not be made with confidence. In the United States, it is standard practice to recover kidneys before acceptance by individual units. On the contrary, in countries such as the United Kingdom and Australia, recovery of kidneys is contingent upon the acceptance and allocation of the kidneys for transplantation [16, 17]. In our study, we have found that donor cancer history was associated with at least a 2-fold greater risk of non-utilization. This finding is consistent with current literature [18], but other characteristics including histological types, stage, prior treatment and duration since cancer treatment, may have influenced the decision-making process. However, these details were not routinely recorded or were inadequately captured in the registries. In addition, the exact reasons of not accepting kidneys from consented donors with a prior cancer history are not collected by ANZDATA and ANZOD registries. We speculate that there may be many reasons including cancer and non-cancer transplant-related factors. However, donors with a prior cancer history who were not consented for organ donation are not routinely captured by the registries and therefore an accurate metric of the total number of donors with a prior cancer history being assessed for possible organ donation is unavailable. The introduction of new OrganMatch clinical data system in Australia from 2019 has allowed the capture of pre-specified reasons for non-utilization of donor organs but these data are not yet available for analysis.

The outcomes of donor cancer transmission in kidney transplantation were summarized and presented in previous literature. In a systematic review of 69 studies (case reports, case series and registry data) of 104 donor-transmitted cancer cases, kidney cancer, melanoma, lymphoma and lung cancer were the four most common donor-transmitted cancers, with less than 1 in 2 kidney transplant recipients surviving beyond 2 years. Donor-transmitted melanoma and lung cancer were associated with the poorest outcome, whereas for donor-transmitted kidney cancer, almost 75% survived beyond 2 years [9]. An updated systematic review in 2020 showed similar findings with donor-transmitted melanoma and lung cancer associated with the poorest recipient prognoses (5 years survival of 43% and 19%, respectively), whereas donor-transmitted kidney cancer and lymphomas had the most favorable recipient prognoses (5 years survival of 93% and 63%, respectively) [8]. A framework for evaluating donors with prior cancers has been proposed by a malignancy subcommittee, part of the *ad hoc* Disease Transmission Advisory Committee (DTAC) of the Organ Procurement and Transplantation Network/United Network for Organ Sharing (OPTN/UNOS). The use of organs from donors with cancers deemed as intermediate (estimated frequency of donor cancer transmission of 1%–10%) or high risk (estimated frequency >10%) are generally not recommended, whereas those at minimal (estimated frequency <0.1%) or low risk (estimated frequency 0.1%–1%) can be considered with appropriate informed consent [19]. Although donors with more aggressive donor cancers such as melanoma, lung cancer and higher stage breast and colon cancers are deemed unsuitable, these risks must be balanced against the projected survival gain for potential recipients. However, the risks of transmission for less common donor cancers are often difficult to define. For example, the risk of transmission of donors with prior central nervous sytem cancers, such as glioblastoma multiforme, is uncertain, with prior studies of such cases showing no conclusive evidence of transmission [3, 5, 20–22]. In our study, of the 141 actual donors with prior solid or haematological cancers (excluding those with NMSCs) whose kidneys were utilized for transplantation, 32% of the donor cancers were gynaecological and prostate cancers, with a lesser proportion of utilized kidneys from donors with prior colorectal, haematological and breast cancers, likely reflecting the general risk tolerance of clinicians when allocating these donor kidneys for transplantation. There were no noticeable differences in the time periods from cancer to organ donation, cancer histological types and treatment for selected donor cancers for intended and actual donors, suggesting that the selection of donors with cancers for consent has been carefully considered. It is important to emphasize that the actual risk of cancer transmission from donor to recipients cannot be determined with certainty in this study because situations where donors with a prior cancer history and did not result in cancer transmission are not captured by the registries. Under-reporting of prior cancer history (of donors) and the possibility of donor cancer transmission is likely. Furthermore, verification of disease transmission using detailed donor-HLA typing or other molecular techniques to ensure the tumour cells are of donor origin are not required by the registries. Consequently, we are unable to provide detailed information regarding the cancer type and the reason of allograft loss from donor cancer in this study.

The evidence that underpinned the current recommendation for donor acceptance are based on case reports and series aligned with expert opinions, and therefore are of low to very low quality. Nevertheless, the decision to accept kidneys from donors with prior cancer history is often contingent on the perceived risks (of potential donor cancer transmission) and benefits (to the potential transplant candidates) of utilizing these kidneys for transplantation. The type, stage, adequacy of prior anti-cancer treatment and follow-up, and the interval from cancer diagnosis to donation must be carefully considered on a case-by-base basis. Specifically, the clinicians will need to balance between the potential risk of “undetected or unrecognised” donor cancer recurrence (and therefore the possibility of donor cancer transmission) against the risk of death without transplantation. However, the recipients’ wish must be respected and informed consent must be obtained. A shared decision making process between the recipients and health professionals, which involve consideration of the patients and their families’ persectives, preferences and circumstances must be valued.

While the precise risk of transmission from donor to recipient of any given cancer is usually unknown, there has been an attempt in broadly categorizing the possible risk of transmission based on the cancer type and stage, its metastatic potential, and its patterns of recurrence in both the transplant and non-transplant setting [19, 23, 24]. It is imperative that all jurisdisctions and donation agencies maintain a register of outcomes of transplants from donors with cancer. These registers should include a well-defined, minimum set of outcomes that are acceptable to patients and may include all cancer characteristics such as tumour histology, stage, cancer genetics and management strategies. Reports on donor transmission events and their outcomes must be published regularly. A global repository that collects high-risk donor details, including those with prior cancer and combined with minimum and real-time data entry requirements with sufficient follow-up periods is essential to inform recommendations to guide informed decisions regarding utilization of these donor organs for transplantation.

This study has a number of potential limitations. Selection, reporting, confounding and information biases are inherent to this observational analysis. These limitations could have hampered accurate estimation of the risk and outcomes of donor transmitted cancers. Inconsistent reporting of follow-up times, the lack of treatment specific details and cancer stage may have precluded the understanding of the actual risk of cancer transmission of organs from donors with prior cancers history with varying clinical, histological, genetic and treatment characteristics.

In this study, we have shown that prior cancer history is a key factor for donor non-utilization. This study also highlights the need to improve data collection relating to the clinical decision-making process of the acceptance and utilization of organs from donors with prior cancer history, as well as the need for accurate records of donor-transmitted cancers in organ transplantation. The establishment of global repositories, combined with minimum and real-time data entry requirements with sufficient follow-up periods are essential to inform recommendations to guide informed decisions regarding utilization of these higher risk donor organs.

## Data Availability

Data extraction from the registry need ethics approval for study investigators to handle the data (as per requirement of the registry’s privacy laws). However, with ethics approval, this or other datasets can be requested from ANZDATA registry. Requests to access the datasets should be directed to request@anzdata.org.au.
