# Supplementary material for: Donors With a Prior History of Cancer: Factors of Non-Utilization of Kidneys for Transplantation
Source: Transpl Int. 2023 Oct 31;36:11883. doi: 10.3389/ti.2023.11883 (PMC10643206; doi:10.3389/ti.2023.11883)
Supplement: Supplementary file 1 [file DataSheet1.PDF]

Supplementary Table 1. Composition of cancer types of "Other" cancers.

Other cancer types	Non-utilized donors (n=21)	Utilized donors (n=17)
Unknown primary site[#]	3	3
Testis	2	2
Tongue	1	1
Lip	0	1
Small intestine	3	1
Stomach	3	0
Pancreas	1	1
Larynx	1	2
Salivary gland	1	0
Other endocrine glands	1	1
Connective or subcutaneous tissue	1	1
Other ill-defined sites	3	1
Nasal cavities and sinuses	0	2
Peritoneum	1	0
Nasopharynx	0	1

[#]For unknown primary cancer, specific cancer types/sites were not specified by reporting centres.

Supplementary Table 2. Association between prior donor cancer history and risk of non-utilization of donor kidneys for transplantation.

**Model of prior cancer including NMSC**	**Non-utilization (OR and 95%CI)**	
**Donor characteristics**	**Consented donors (intended/actual)**	**Actual donors**
Prior cancer	2.29 (1.68, 3.13)	2.36 (1.58, 3.53)
Age (per 10-year increase)	1.11 (1.04, 1.18)	1.11 (1.02, 1.21)
Hypertension	1.72 (1.41, 2.09)	2.16 (1.67, 2.80)
Smoking history		
None	1.00	1.00
Former	0.93 (0.74, 1.16)	0.83 (0.62, 1.12)
Current	1.40 (1.16, 1.70)	1.07 (0.83, 1.39)
Diabetes	2.08 (1.58, 2.73)	1.94 (1.38, 2.74)
Ethnicity		
White	1.00	1.00
Australian Aboriginal/TSI	2.18 (1.23, 3.85)	2.55 (1.23, 5.30)
Asians	1.77 (1.26, 2.49)	1.82 (1.18, 2.81)
New Zealand Māori	1.15 (0.50, 2.62)	1.37 (0.48, 3.86)
Others	1.62 (0.90, 2.91)	2.43 (1.25, 4.74)
Positive Hepatitis C virus NAT	84.49 (34.12, 209.22)	101.36 (36.76, 279.50)

**Donation pathway characteristics**		
DCDD (vs. DNBD)	5.32 (4.46, 6.34)	1.73 (1.33, 2.26)
Donor terminal eGFR (per 10mL/min/1.73m^2 increase)	0.88 (0.86, 0.91)	0.85 (0.82, 0.87)
Era		
1989-1998	1.00	1.00
1999-2007	0.74 (0.44, 1.23)	0.7 (0.46, 1.31)
2008-2017	4.21 (2.79, 6.35)	2.68 (1.74, 4.12)
**Model of prior cancer excluding NMSC**	**Non-utilization (OR and 95%CI)**	
**Donor characteristics**		
Prior cancer	2.33 (1.59, 3.41)	2.53 (1.57, 4.08)
Age (per 10-year increase)	1.12 (1.05, 1.19)	1.12 (1.03, 1.22)
Hypertension	1.72 (1.41, 2.09)	2.31 (1.16, 4.60)
Smoking history		
None	1.00	1.00
Former	0.93 (0.74, 1.17)	0.84 (0.62, 1.13)
Current	1.40 (1.15, 1.70)	1.07 (0.83, 1.39)
Diabetes	2.07 (1.58, 2.72)	1.94 (1.37, 2.74)
Ethnicity		

White	1.00	1.00
Australian Aboriginal/TSI	2.14 (1.21, 3.79)	2.50 (1.20, 5.19)
Asians	1.73 (1.23, 2.43)	1.78 (1.15, 2.75)
New Zealand Māori	1.13 (0.50, 2.58)	1.34 (0.48, 3.80)
Others	1.58 (0.88, 2.85)	2.38 (1.22, 4.64)
Positive Hepatitis C virus NAT	84.73 (34.22, 209.76)	100.60 (36.45, 277.69)
**Donation pathway characteristics**		
DCDD (vs. DNBD)	5.35 (4.48, 6.37)	1.74 (1.33, 2.27)
Donor terminal eGFR (per 10mL/min/1.73m^2 increase)	0.88 (0.86, 0.90)	0.85 (0.82, 0.87)
Era		
1989-1998	1.00	1.00
1999-2007	0.74 (0.44, 1.23)	0.78 (0.46, 1.31)
2008-2017	4.30 (2.85, 6.48)	2.75 (1.79, 4.22)

Data expressed as adjusted odds ratio (OR) and 95% confidence intervals (95%CI). TSI – Torres Strait Islander, DCDD – donation after circulatory determination of death, DNBD, donation after neurological determination of death, NAT – nucleic acid test, eGFR – estimated glomerular filtration rate.

Table 3. Cancer types and characteristics of intended and actual donors with prior cancer.

	Intended donors (non-utilized) n=86	Actual donors (non-utilized) n=62	Actual donors (utilized) n=197	p-values
**Donor characteristics**				
**Age (mean [95%CI])**	57.4 (53.9, 60.9)	61.1 (57.6, 64.5)	56.9 (55.2, 58.6)	0.108
**Female (n, %)**	35 (40.7)	31 (50.0)	107 (54.3)	0.109
**Years from cancer to donation (mean [95%CI])**	10.3 (7.4, 13.2)	6.5 (4.1, 8.8)	11.0 (9.0, 13.0)	0.064
**Diabetes (n, %)**	6 (7.0)	13 (21.0)	13 (6.6)	0.012
**Hypertension (n, %)**	35 (40.7)	33 (53.2)	69 (35.0)	0.086
**Specific donor cancer types**				
**Melanoma (n)**	17	5	18	
**Sites (n)**				
**Skin**	15	5	16	
**Non-skin**	0	0	1	
**Unknown**	2	0	1	
**Treatment**				
**Surgery**	14	4	14	
**None/Others**	1	0	2	
**Unknown**	2	1	2	
**Years to donation[#]**	7.1 (5.1, 15.6)	23.5 (11.3, 30.2)	10.1 (5.4, 28.5)	0.10
**Brain (n)**	9	3	19	
**Types**				
**Astrocytoma**	4	0	8	
**Low-grade glioma**	0	1	2	
**High grade glioma/GBM**	1	2	5	
**Medulloblastoma**	3	0	0	
**Meningioma**	1	0	0	
**Others**	0	0	4	
**Treatment**				
**Surgery**	7	2	5	
**Radiotherapy**	1	0	1	

Chemotherapy	0	0	1	
None	1	1	9	
Unknown/others	0	0	3	
Years to donation#	3.2 (0.6, 26.8)	0.1 (0.0, 12.7)	0.7 (0.1, 8.8)	0.26
**Colorectal (n)**	5	6	3	
Types				
Adenocarcinoma	3	2	2	
Unknown	1	1	0	
Carcinoid	0	1	0	
Others	1	2	1 (carcinoma-in-situ)	
Treatment				
None	0	2	0	
Surgery	4	2	3	
Chemotherapy/radiotherapy	0	0	0	
Unknown/others	1	2	0	
Years to donation#	6.4 (4.9, 8.0)	8.8 (0.02, 14.3)	14.1 (10.1, 26.7)	0.32
**Breast (n)**	3	4	14	
Types				
Adenocarcinoma	2	2	2	
Invasive ductal carcinoma	1	1	2	
Ductal carcinoma in situ	0	1	1	
Unknown	0	0	9	
Treatment				
None	1	0	0	
Surgery	2	2	11	
Chemotherapy/radiotherapy	0	1	1	
Unknown/others	0	1	2	
Years to donation#	9.4 (3.7, 31.1)	13.4 (7.5, 19.1)	20.3 (17.8, 25.3)	0.35
**Prostate (n)**	8	3	14	
Types				
Adenocarcinoma	6	0	12	
Others	1	2	0	
Unknown	1	1	2	

**Treatment**				
**None**	1	1	1	
**Surgery**	4	2	10	
**Chemotherapy/radiotherapy**	1	0	2	
**Unknown/others**	2	0	1	
**Years to donation#**	5.3 (0.3, 9.0)	2.8 (0.2, 7.5)	5.4 (3.3, 11.0)	0.31
**Kidney/bladder (n)**	1	9	11	
**Types**				
**RCC**	0	7	8	
**Papillary cancer (kidney)**	0	1	0	
**Kidney oncocytoma**	0	1	1	
**Bladder (urothelial/TCC)**	1	0	2	
**Treatment**				
**None**	0	9	8	
**Surgery**	1	0	2	
**Unknown/others**	0	0	1	
**Years to donation#**	2.0 (-,-)	-	0.8 (0.3, 1.1)	0.16
**Gynaecological (n)**	5	3	30	
**Types**				
**Cervical cancer (SCC/adenocarcinoma)**	4	1	13	
**Cervical cancer in situ**	0	0	0	
**Uterine**	0	0	2	
**Others/Unknown**	1	2	15	
**Treatment**				
**None**	0	0	1	
**Surgery**	2	2	22	
**Chemotherapy/radiotherapy**	0	1	0	
**Unknown/others**	3	0	7	
**Years to donation#**	20.4 (5.8, 26.8)	22.4 (13.2, 22.8)	11.0 (6.4, 18.1)	0.39
**Haematological (n)**	3	3	6	
**Types**				
**Leukaemia**	1	0	2	
**Lymphoma**	2	3	4	

**Treatment**				
**None**	1	2	3	
**Surgery**	0	0	0	
**Chemotherapy/radiotherapy**	1	0	2	
**Unknown/others**	0	1	1	
**Years to donation#**	1.3 (0.5, 24.3)	-	3.6 (0.8, 16.5)	0.65

Data expressed as number (%), mean (95% confidence interval [95%CI]) or as median (interquartile range [IQR]). #Represents median (IQR) years to donation using available recorded data. GBM – glioblastoma multiforme, RCC – renal cell cancer, DCIS – ductal carcinoma-in-situ, TCC – transitional cell cancer.

Supplementary Table 4. Characteristics of kidney transplant recipients reported to experience potential donor cancer-related allograft loss from deceased donors without a prior history of cancer at the time of organ donation.

Cases of potential donor cancer transmission (Year of transplant)	Donor age	Recorded causes of donor death	Time to allograft loss in days*	Time to death in days (recorded causes of death)
**1 (1992)**	45	Hypoxia	8	1058 (infection)
**2 (1992)[#]**	50	Intracranial haemorrhage	304	474 (cardiac)
**3 (1992)[#]**	50	Intracranial haemorrhage	356	3248 (cancer - melanoma)
**4 (1996)**	55	Intracranial haemorrhage	2	Living^
**5 (1996)**	67	Traumatic brain injury	0	Living^
**6 (1997)**	75	Hypoxia (respiratory arrest)	102	4818 (adenocarcinoma in unknown site)
**7 (2001) [#]**	59	Intracranial haemorrhage	3	Living^
**8 (2001) [#]**	59	Intracranial haemorrhage	4	4051 (cardiac)
**9 (2010)**	61	Hypoxia (cardiac arrest)	1095	Living^
**10 (2010)**	69	Intracranial haemorrhage	852	2737 (unknown)
**11 (2014)**	72	Intracranial haemorrhage	108	Living^
**12 (2015)**	56	Traumatic brain injury	2	Living^

*[#]Same donor. *All causes of allograft loss reported (to the ANZDATA registry) to be attributed to potential donor cancer. ^Reported to be alive at the end of December 2017.*
